# Salivary IGF-1 and Alkaline Phosphatase-Based Modeling for Skeletal Maturity Prediction in Orthodontic Patients

**DOI:** 10.1155/2022/2390865

**Published:** 2022-01-21

**Authors:** Asma Sookhakian, Maryam Zahed, Hamidreza Pakshir, Shabnam Ajami

**Affiliations:** ^1^Student Research Committee, School of Dentistry, Shiraz University of Medical Sciences, Shiraz, Iran; ^2^Oral and Dental Disease Research Center, Department of Oral and Maxillofacial Medicine, School of Dentistry, Shiraz University of Medical Sciences, Shiraz, Iran; ^3^Orthodontics Research Center, School of Dentistry, Shiraz University of Medical Sciences, Shiraz, Iran

## Abstract

**Results:**

A strong positive correlation was found between CA and cervical stages (*r* = 0.836, *P* < 0.001). Based on the regression model analysis, the model which combined IGF-1, ALP, and CA provided the best prediction at *P* < 0.001 with McFadden's pseudo *R*^2^ value of 0.552 for cervical stage prediction and 0.646 for growth phase prediction. In particular, its predictive ability for the prepubertal, pubertal, and postpubertal growth phases was 95%, 80%, and 90%, respectively.

**Conclusions:**

A new model with the combination of salivary IGF-1 and ALP with CA can be used as a less invasive method to determine the cervical stage and also growth phase with high predictive ability in healthy subjects.

## 1. Introduction

One orthodontic treatment objective is to treat skeletal discrepancies by utilizing the skeletal growth potential [1]. Growth potential is defined as the increase that may occur between current and final bone size [2]. During growth phases, the skeletal tissue of the maxillomandibular complex is changing due to sutures and osteogenic cartilage proliferation depending on the complex interactions of many genes, hormones, growth factors, and the environment [3, 4]. Therefore, extrinsic factors including orthopedic and functional treatment modalities can be used to modify the amount and direction of facial growth [3].

The term maturation refers to a developmental process that completes physical growth [2]. Particularly, skeletal maturity is a stage of bone development [5]. The parameters that are proposed as diagnostic aids for skeletal maturity assessment are chronological age (CA), stature height, pubertal markers, tooth eruption and dental maturation, and radiographic assessment of bone [6–8].

The skeletal maturity assessment using radiographs is widely used to predict the pubertal growth phase and to estimate the proportion of remaining growth [2, 6]. During growth, certain bones demonstrate an organized ossification that can be seen radiologically. Although the changes can be observed in hand, elbow, shoulder, cervical vertebrae, hip, knee, and foot, skeletal maturity assessment based on the hand-wrist and cervical vertebrae has been developed [9]. There has been an interest in maturational changes in size and shape of the cervical vertebrae from the first decades of the twentieth century [10]. Several cervical vertebral maturation (CVM) methods have been proposed, and some modifications and improvements are available [11]. Although recent studies have shown that the CVM method might be more beneficial than hand-wrist radiographs because it eliminates the second dose of radiation [12], there are some limitations in CVM staging: (1) significant errors in clinical usage due to observers' subjective judgment of morphologic changes in cervical vertebrae [11]; (2) not accurately signaling the onset of the peak in mandibular growth; (3) difficulty in visualizing the subtle changes in the vertebrae due to incorrect neck posture while taking the radiograph [13]; (4) variability in cervical area of vertebral column due to different skeletal relationship of the jaws, body posture, and shapes of facial components; (5) fusion of second and third cervical vertebrae in 14.3% of healthy subjects [14]; (6) not suggesting X-ray exposure more than once a year because of additional radiation exposure hazards [15]. Nowadays, newer possibilities to assess the skeletal maturity are offered by bone formation and resorption biomarkers that change with bone growth and remodeling. Previous cross-sectional studies on biomarkers including insulin-like growth factor-1 (IGF-1) and alkaline phosphatase (ALP) yielded encouraging results [15–22]. Therefore, the present study is aimed at investigating further and developing models for prediction of cervical vertebral maturational stage and also growth phase using CA, salivary IGF-1, and ALP for the first time.

## 2. Subjects and Methods

In this cross-sectional study, orthodontic patients referred to the Dental School of Shiraz University of Medical Sciences in the spring of 2021 were included. All subjects were included according to the following criteria: (1) healthy subjects (both males and females) in the age range of 7 to 20 years with documented birth dates; (2) subjects having an acceptable lateral cephalogram taken in the last 6 months for orthodontic evaluation; (3) visibility of the second, third, and fourth vertebrae in all films. Exclusion criteria were radiographs of poor quality, history of trauma or operation in the area of the cervical vertebrae, systemic disease, growth abnormalities, nutritional problems, xerostomia, self-reported pregnancy or lactation, and taking medications in the last month preceding entry to the study.

Subjects deemed eligible were scheduled for participation in the study after obtaining an informed consent from either themselves or their legal guardians. The study was conducted after approval by the ethical committee of Shiraz University of Medical Sciences (IR.SUMS.DENTAL.REC.1399.197). Saliva samples for all subjects were collected at the same time of year (spring) and in the morning between 8:00 and 11:00 AM. The subjects were instructed to refrain from drinks, food, chewing gum, and tooth brushing at least 90 minutes before sample collection. In addition, they were asked to rinse their mouth with plain water and rest for 5 minutes. Then, unstimulated whole saliva was collected by the spitting method [23].

The IGF-1 level was quantitatively determined by Enzyme-Linked Immunosorbent Assay (ELISA) using the Human IGF-1 ELISA Kit (ZellBio GmbH, Germany), following the manufacturer's instruction. The optical density (OD) was read on a microplate reader at 450 nm wavelength. Finally, the IGF-1 level was determined in ng/ml. The ALP level was quantitatively determined based on the DGKC (the Standard of German Society for Biochemistry) using the Human ALP Kit (Biorexfars, Iran) with the help of an autoanalyzer at 405 nm wavelength, following the manufacturer's instruction. The value of ALP level was expressed in unit/l.

All the subjects were grouped according to Baccetti's 6-stage CVM method [7]. In this method, two sets of variables were analyzed: (1) presence or absence of a concavity at the lower border of the body of C_2_, C_3_, and C_4_; (2) morphologic shape of the body of C_3_ and C_4_ (trapezoid, rectangular horizontal, square, and rectangular vertical). To gain additional information, the sample was divided into three groups on the basis of growth phase according to Perinetti et al. [24]: prepubertal (CS1 and CS2), pubertal (CS3 and CS4), and postpubertal (CS5 and CS6). For instance, CS1 and CS2 were combined and considered as the prepubertal growth phase. All the lateral cephalograms were assessed independently by two examiners (one orthodontist and one oral and maxillofacial radiologist) who were blind to the other information regarding each case. A third examiner was consulted in case of disagreement. In this regard, the results recorded by two examiners were compared to that of the third examiner and the similar results obtained by two examiners were recorded as the determined stage.

Statistical Package for the Social Sciences (SPSS) software was used to perform statistical analysis. Descriptive statistics were used to measure the frequency, percentage, minimum (Min), maximum (Max), lower bound (LB), upper bound (UB), mean, and standard deviation (SD). The Cohen's Kappa statistic was used to measure interexaminer reliability for CVM staging. The significance of the difference in the sample distribution of different cervical stages was evaluated by the chi-square test. This test was also used to analyze the balancing of the groups by sex. The Shapiro-Wilk test was used to test the normality of the distribution of the variables. Since data follows normality, the independent samples *t*-test was used to compare the mean CA values between the sexes in each cervical stage. The Pearson correlation coefficient was calculated among CA values and cervical stages.

Seven multinomial logistic regression models were used to predict the cervical stage based on salivary IGF-1 level, salivary ALP level, and CA. We denoted a model by its predictors: for instance, (CA+ALP) having CA and ALP effects. In other words, the explanatory variables are CA and salivary ALP level. In this study, the adequacy of fit for a logistic regression model was assessed by the significance of the chi-square test of the model coefficients, which assesses the decrease in the log-likelihood (LL) of the regression model containing the full set of predictors when it is compared to the model that contains only the intercept and determines whether the former significantly improves prediction over the latter. Then, McFadden's pseudo *R*^2^ (*R*^2^_MF_) was used to assess the predictive ability of each model. It is one minus the ratio of the full-model LL to the intercept-only LL [25].

(1) $$\begin{array}{c} R_{\text{MF}}^{2}=1-\frac{\text{LL}\left(\text{Full}\right)}{\text{LL}\left(\text{Null}\right)}. \end{array}$$

According to Mokhtarian, McFadden's pseudo *R*^2^ of 0.3 is indicative of a decent model fit [26]. The overall correct classification rate was also measured in all models.

The logistic regression model for a binary response variable *Y* and an explanatory variable *X*, *π*(*x*) = *P*(*Y* = 1 | *X* = *x*) = 1–*P*(*Y* = 0 | *X* = *x*), is

(2) $$\begin{array}{c} \pi \left(x\right)=\frac{\exp\;\left(\alpha +\beta x\right)}{1+\exp\;\left(\alpha +\beta x\right)}. \end{array}$$

Equivalently, the log odds, called the logit, have the linear relationship.

(3) $$\begin{array}{c} \text{Logit}\left[\pi \left(x\right)\right]=\text{Log}\frac{\pi \left(x\right)}{1-\pi \left(x\right)\;}=\alpha +\beta x. \end{array}$$

*β* determines whether *π*(*x*) is decreasing or increasing as *x* increases. Exponentiation on both sides of (3) shows that the odds are an exponential function of *x*. This provides a basic interpretation for the magnitude of *β*: for every 1-unit increase in *x*, the odds increase multiplicatively by  *e*^*β*^ . In other words, *e*^*β*^  is an odds ratio, the odds at *X* = *x* + 1 divided by the odds at *X* = *x* [27].

In the present study, *Y* was a categorical response. Our multicategory logit models for nominal response variables simultaneously describe log odds for all (*J*/2) pairs of categories. Given a certain choice of *J* − 1 of these, the rest are redundant.

Logit models of this study paired each response category with the last category (CS6 and postpubertal growth phase) as the baseline category. The model

(4) $$\begin{array}{c} \text{Log}\frac{\pi j\left(x\right)}{\pi J\left(\text{x}\right)}=\alpha j+\beta^{\prime }j\;x,\;j=1,\cdots ,J-1, \end{array}$$

described the effects of *x* on these *J* − 1 logits. The *J* − 1 equations determine parameters for logits with other pairs of response categories, since

(5) $$\begin{array}{c} \text{Log }\frac{\pi a\left(x\right)}{\pi b\left(x\right)}=\text{Log }\frac{\pi a\left(x\right)}{\pi J\left(x\right)}-\text{Log }\frac{\pi b\left(x\right)}{\pi J\left(x\right)}\left(27\right). \end{array}$$

The equation that expresses multinomial logit models directly in terms of response probabilities { *π* *_j_*(*x*)} is

(6) $$\begin{array}{c} \pi_{j}\left(x\right)=\frac{\exp\left(\alpha j+\beta^{\prime }j\;x\right)}{1+\sum_{h=1}^{J-1}\exp\left(\alpha h+\beta^{\prime }h\;x\right)}. \end{array}$$

The parameters equal to zero for the baseline category for identifiable reasons (*α*_*J*_ = 0 and *β*_*J*_ = 0). The denominator of (6) is the same for each *j*. The numerators for various *j* sum to the denominator, so ∑*j* *π* *_j_*(*x*) = 1 [27].

For all analysis, *P* value < 0.05 was considered statistically significant.

## 3. Results

From 84 subjects with sufficient radiographs, 55 patients were selected (both female and male) in the age range of 7.0 to 20.0 years. Three subjects had insufficient saliva samples, 8 could not participate due to lack of time, and 18 were unwilling to participate due to COVID-19 pandemic. The study sample consisted of CS1 (*N* = 9), CS2 (*N* = 11), CS3 (*N* = 9), CS4 (*N* = 6), CS5 (*N* = 9), and CS6 (*N* = 11). The kappa statistic for interexaminer reliability was 0.759. Sample distribution among the cervical stages was statistically uniform (*P* = 0.871). In addition, sex distribution of the sample in each stage and also in total was uniform (*P* = 0.500).

**Figure 1:**
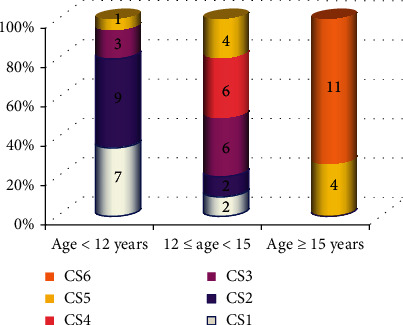
Distribution of cervical stages in different age groups.

**Figure 2:**
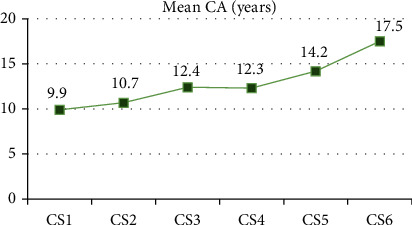
Chronological age (CA) trend in relation to the cervical stages.

Table 1 summarizes the descriptive analysis of age for each cervical stage. The distribution of cervical stages in three different age groups is presented in Figure 1. From this figure, we can note that 80% of under 12 age group was in CS1 and CS2 (prepubertal), 60% of 12-15 age group was in CS3 and CS4 (pubertal), and all the subjects of 15 age group and above were in CS5 and CS6 (postpubertal).  Figure 2 also indicates graphically the trend of mean CA in relation to cervical stages. In the present study, there was a strong positive correlation between CA and cervical stages (*r* = +0.836, *P* < 0.001).

**Figure 3:**
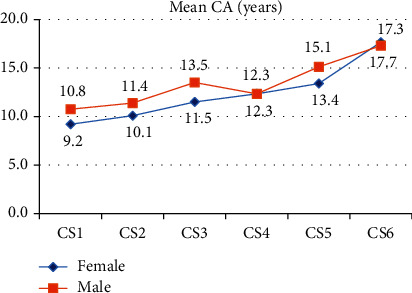
Chronological age (CA) trend in female and male groups in relation to the cervical stages.

Upon comparing the male and female groups, we found that the mean CA of females was significantly lower than males at CS2 and CS3 (*P* < 0.05). As shown in Figure 3, the mean differences were 1.3 years at CS2 and 2 years at CS3.

This study presented a saliva-based modeling for both cervical stage and growth phase prediction. In this regard, seven multinomial logistic regression models, each using one or more explanatory variables, were used. The explanatory variables were statistically significant (*P* < 0.05) in all of the models.  Table 2 contains fit statistics of seven multinomial logistic regression models for cervical stage prediction. Thereafter, the sample was divided into three groups on the basis of growth phase (prepubertal, pubertal, and postpubertal) and the fit statistics of these seven multinomial logistic regression models were also assessed for growth phase prediction (Table 3).

It is important to mention that the ability of these models was higher in growth phase prediction compared to the cervical stage prediction. The highest predictive ability (overall correct classification rate) was related to Model 7, which reached 70.9% for cervical stage prediction and 89.1% for growth phase prediction. In more detail, the ability of this model for correct prediction of different growth phases is presented in Table 4.

Model 7 which had the highest predictive ability compared to the other models described the effects of CA, IGF-1, and ALP on prediction of skeletal maturity. Results of this regression model for predicting cervical stage and growth phase are summarized in Tables 5 and 6, respectively.

It is important to note that the coefficient *β*_*j*_ represents the change in the log odds that would result from a one unit change in one variable when all the other variables are fixed. For example, *β*_1_ CA is the change in Log(*π*_CS1_/*π*_CS6_) corresponding to one unit change in CA, when IGF-1 and ALP are fixed. The multivariable equations generated from these tables can be used to

- determine parameters for logits with other pairs of response category. For instance, for (*π*_CS2_/*π*_CS4_),

(7) $$\begin{array}{c} \text{Log }\left(\pi_{\text{CS}2}/\pi_{\text{CS}4}\right)=\text{Log }\left(\pi_{\text{CS}2}/\pi_{\text{CS}6}\right)-\text{Log }\left(\pi_{\text{CS}4}/\pi_{\text{CS}6}\right)=\left(46.951-3.437\text{ CA}–0.334\text{ IGF}–0.001\text{ ALP}\right)-\left(29.901-2.180\text{ CA}+0.386\text{ IGF}+0.031\text{ ALP}\right)=17.050–1.257\text{ CA}–0.720\text{ IGF}–0.032\text{ ALP} \end{array}$$

(ii) estimate odds ratios, exp (*β*_*j*_), by identifying pairs of response category (e.g., for a given CA and ALP, the estimated odds that individuals be on CS3 instead of CS6 increase by exp (3.041) = 20.93 for every 1-unit increase in IGF)
(iii) determine the response probabilities {*π*_*j*_(x) = exp(*αj* + *βj* CA + *βj* IGF + *βj* ALP)/(1 + ∑_*h*=1_^*J*−1^exp(*αh* + *βh*CA + *βh*IGF + *βh*ALP)). For example, the estimated probabilities that an individual with CA = 12, IGF = 2.3, and ALP = 45 be at CS3 is 0.8706

## 4. Discussion

Despite the fact that CA have the advantage of being accessible and inexpensive in terms of application [28], genetics, hormones, nutrition, race, and environment may lead to an inconsistency between the CA and physiological maturity. Nevertheless, growth rate changes of adolescents can be well monitored if a reliable CA determination based on physiological parameters is guaranteed [29]. The term biomarker refers to any substance, structure, process, or its products that can be measured in the body and influence or predict the incidence of outcome or disease [30]. They can be measured from various biologic fluids [31] such as blood, urine, gingival crevicular fluid, and saliva. Salivary biomarkers avoid X-ray exposure and assess the skeletal maturity in an objective manner. Moreover, smaller sample fraction, good patient compliance, easy collection (without specialized equipment or personnel), storage and transportation, cost effectiveness (even for the screening of large populations), greater sensitivity, and correlation with levels in blood are the other advantages that make saliva sampling very attractive to several researchers [32, 33].

The present study initially demonstrated a strong positive correlation between CA and cervical stages. Even though this result differs from two published studies [34, 35] that showed a marked CA variation for skeletal maturity, this is consistent with previous results [36–39] that reported a significant positive correlation between CA and skeletal maturity. Upon comparing female and male groups, we found that the females preceded the males 1.3 years at CS2 and 2 years at CS3. Therefore, the present study confirmed previous studies [36, 38] suggesting that females are more advanced than males in skeletal maturation.

The goal of the present study was to predict the cervical stage and growth phase based on the salivary IGF-1 level, salivary ALP level, and CA for the first time. In this regard, seven multinomial logistic regression models, each using one or more explanatory variables, were compared. As shown in Tables 2 and 3, the predictive ability (overall correct classification rate) of IGF-1 was higher than ALP but lower than CA in isolation. We found much higher value for predictive ability of binary and ternary combinations of them. Our findings indicated that the *R*^2^_MF_ for all models including CA (Model 1, Model 4, Model 5, and Model 7) was above 0.3, indicating a decent model fit. Therefore, CA is a high predictor of skeletal maturity and combining biomarkers (salivary IGF-1 and/or ALP) with CA can also improve predictive ability of the regression models. The current study results showed that the highest predictive ability was related to Model 7 (CA+IGF-1+ALP). Indeed, Model 7 which combined CA, IGF-1, and ALP provided the best prediction compared to the other models, which reached 70.9% for cervical stage prediction and 89.1% for growth phase prediction. In particular, its predictive ability for the prepubertal, pubertal, and postpubertal growth phases were 95%, 80%, and 90%, respectively. We can be confident in accepting our positive results as being significant predictors because the statistics account for possible type *Ι* error. Therefore, the notably large predictive ability of the seventh model, especially for the prepubertal growth phase, might be responsible for the quantification of salivary IGF-1 and ALP levels in growing healthy subjects in clinical practice. The multivariable prediction equations derived in the present study (Tables 5 and 6) can be used to predict the cervical stage and also growth phase of a healthy subject clinically by the use of a simple computing software.

There are a few studies which have used biomarkers to predict skeletal maturity. The findings observed in this study mirror those of a previous study [39] that have shown that the use of biomarkers in combination with CA can enhance skeletal maturity assessment. In that study, they increased the amount of ability of CA for cervical stage prediction from 51.9% to 53.2% with the addition of salivary ALP. However, we were able to increase the amount of predictive ability in our models to 70.9% with the addition of two biomarkers including salivary IGF-1 and ALP. In another previous study on modeling for skeletal maturity prediction [28], a multinomial logistic regression model was used to predict the growth phase based on salivary B-ALP level, BMI percentile, and CA. Results of their study indicated that the ability of this model for correct prediction of prepubertal, pubertal, and postpubertal growth phases was 78%, 57.7%, and 81.4%, which were lower compared to those in our study (Table 4). Thus, the models observed in our study which combine CA with ALP and IGF-1 seem to be the best models presented so far in literature.

To note, in our study, the saliva collection was not achieved exactly at the same time of radiographic exposure due to ethical considerations. However, only subjects who had lateral cephalograms within the last 6 months were included in the present study, following a similar approach as in a previous study [39]. Future studies on the current topic are suggested in examining more variables that can play a significant role in skeletal maturity assessment.

## 5. Conclusion

This study is the first that investigated the effects of salivary IGF-1, salivary ALP, and CA on prediction of cervical vertebral maturational stages using regression models and determined the prediction equations. In conclusion, the method which combined salivary IGF-1 and ALP levels with CA can be used to determine the cervical stage and also growth phase with high predictive ability in healthy subjects.

## Figures and Tables

**Table 1:** Chronological age (CA) descriptive statistics at different cervical stages (*n* = 55).

Cervical stage	*N*	CA (years)		
		Mean ± SD	Min-Max	95% CI for mean (LB-UB)
1	9	9.89 ± 1.88	7.0–12.5	8.44-11.34
2	11	10.68 ± 0.96	9.0-12.0	10.04–11.32
3	9	12.39 ± 1.54	10.0–14.0	11.21–13.57
4	6	12.33 ± 0.52	12.0–13.0	11.79–12.88
5	9	14.17 ± 1.98	11.0–17.5	12.64–15.69
6	11	17.50 ± 1.80	15.0-20	16.29–18.71

LB = lower bond; UB = upper bond.

**Table 2:** Fit statistics of multinomial logit models for cervical stage prediction.

Model	Likelihood-ratio chi-square statistic (*P* value)	df	*R* ^2^_MF_	Correct classification rate (%)
Model 1: (CA)	74.653 (<0.001)^∗^	5	0.383	50.9
Model 2: (IGF-1)	28.087 (<0.001)^∗^	5	0.144	38.2
Model 3: (ALP)	22.335 (<0.001)^∗^	5	0.114	34.5
Model 4: (CA+IGF-1)	91.958 (<0.001)^∗^	10	0.471	58.2
Model 5: (CA+ALP)	91.784 (<0.001)^∗^	10	0.470	65.5
Model 6: (IGF-1+ALP)	46.484 (<0.001)^∗^	10	0.238	47.3
Model 7: (CA+IGF-1+ALP)	107.802 (<0.001)^∗^	15	0.552	70.9

^∗^ *P* < 0.05 considered significant.

**Table 3:** Fit statistics of multinomial logit models for growth phase prediction.

Model	Likelihood-ratio chi-square statistic (*P* value)	df	*R* ^2^_MF_	Correct classification rate (%)
Model 1: (CA)	59.493 (<0.001^∗^)	2	0.496	76.4
Model 2: (IGF-1)	21.184 (<0.001^∗^)	2	0.177	60
Model 3: (ALP)	14.135 (=0.001^∗^)	2	0.118	56.4
Model 4: (CA+IGF-1)	69.888 (<0.001^∗^)	4	0.583	80
Model 5: (CA+ALP)	69.067 (<0.001^∗^)	4	0.576	83.6
Model 6: (IGF-1+ALP)	30.526 (<0.001^∗^)	4	0.255	61.8
Model 7: (CA+IGF-1+ALP)	77.497 (<0.001^∗^)	6	0.646	89.1

^∗^ *P* < 0.05 considered significant.

**Table 4:** Correct classification rates of Model 7 for growth phase prediction.

Observed	Predicted			Correct classification rate (%)
	Prepubertal	Pubertal	Postpubertal	
Prepubertal	19	1	0	95
Pubertal	1	12	2	80
Postpubertal	1	1	18	90

**Table 5:** Estimated parameters in Model 7 for cervical stage prediction.

Logit	*j*	Intercept (*α*_*j*_)	(*Β*_*j*_)		
			CA	IGF-1	ALP
Log (*π*_CS1_/*π*_CS6_)	1	56.235	-3.918	-3.101	-0.035
Log (*π*_CS2_/*π*_CS6_)	2	46.951	-3.437	-0.334	-0.001
Log (*π*_CS3_/*π*_CS6_)	3	22.738	-2.364	3.041	0.154
Log (*π*_CS4_/*π*_CS6_)	4	29.901	-2.180	0.386	0.031
Log (*π*_CS5_/*π*_CS6_)	5	22.605	-1.371	-1.409	0.020

^∗^The baseline category was CS6 (*J* = 6).

**Table 6:** Estimated parameters in Model 7 for growth phase prediction.

Logit	*j*	Intercept (*α*_*j*_)		(*Β*_*j*_)	
			CA	IGF-1	ALP
Log (*π*_Prepubertal_/*π*_Postpubertal_)	1	29.002	-2.318	0.437	-0.035
Log (*π*_Pubertal_/*π*_Postpubertal_)	2	8.207	-1.026	2.390	0.054

^∗^The baseline category was postpubertal growth phase (*J* = 3).

## Data Availability

The data supporting the findings of this study are available upon reasonable request from the corresponding author. However, restrictions were applied to the public availability of these data, because of the patient's confidentiality.

## References

[1] Santiago R. C., De Miranda Costa L. F., Vitral R. W. F., Fraga M. R., Bolognese A. M., Maia L. C. (2012). Cervical vertebral maturation as a biologic indicator of skeletal maturity. *Angle Orthodontist*.

[2] Araujo M. T. S., Cury-Saramago A. A., Motta A. F. J. (2011). Guias clínicos e radiográficos utilizados para a predição do surto de crescimento puberal. *Dental Press Journal of Orthodontics*.

[3] Makaremi M., Lacaule C., Mohammad-Djafari A. (2019). Deep learning and artificial intelligence for the determination of the cervical vertebra maturation degree from lateral radiography. *Entropy*.

[4] Tripathi T., Gupta P., Rai P. (2017). Biochemical markers as skeletal maturity indicators. *International Journal of Orthodontic Rehabilitation*.

[5] Bahreman A. (2013). *Early-age orthodontic treatment*.

[6] Flores-Mir C., Nebbe B., Major P. W. (2004). Use of skeletal maturation based on hand-wrist radiographic analysis as a predictor of facial growth: a systematic review. *Angle Orthodontist*.

[7] Baccetti T., Franchi L., McNamara J. A. (2005). The cervical vertebral maturation (CVM) method for the assessment of optimal treatment timing in dentofacial orthopedics. *Seminars in Orthodontics*.

[8] Perinetti G., Contardo L., Gabrieli P., Baccetti T., Di Lenarda R. (2012). Diagnostic performance of dental maturity for identification of skeletal maturation phase. *The European Journal of Orthodontics*.

[9] Dhiman S., Maheshwari S., Verma S. K. (2015). Assessment of maturity in orthodontics: a review. *Journal of Advanced Clinical and Research Insights*.

[10] Baccetti T., Franchi L., McNamara J. A. (2002). An improved version of the cervical vertebral maturation (CVM) method for the assessment of mandibular growth. *Angle Orthodontist*.

[11] Zhao X. G., Lin J., Jiang J. H., Wang Q., NG S. H. (2012). Validity and reliability of a method for assessment of cervical vertebral maturation. *Angle Orthodontist*.

[12] Jaqueira L. M. F., Armond M. C., Pereira L. J., de Alcântara C. E. P., Marques L. S. (2010). Determining skeletal maturation stage using cervical vertebrae: evaluation of three diagnostic methods. *Brazilian Oral Research*.

[13] Gupta S., Deoskar A., Gupta P., Jain S. (2015). Serum insulin-like growth factor-1 levels in females and males in different cervical vertebral maturation stages. *Dental Press Journal of Orthodontics*.

[14] Tripathi T., Gupta P., Rai P., Sharma J., Gupta V. K., Singh N. (2019). Longitudinal evaluation of the association between insulin-like growth factor-1, bone specific alkaline phosphatase and changes in mandibular length. *Scientific Reports*.

[15] Jain N., Tripathi T., Gupta S. K., Rai P., Kanase A., Kalra S. (2017). Serum IGF-1, IGFBP-3 and their ratio: potential biochemical growth maturity indicators. *Progress in Orthodontics*.

[16] Ryan J., Mantle T., Costigan D. C. (1992). A normal population study of human salivary insulin-like growth factor 1 (IGF 1) concentrations from birth through puberty. *The Journal of Clinical Endocrinology & Metabolism*.

[17] Ishaq R., Soliman S., Foda M. Y., Fayed M. M. S. (2012). Insulin-like growth factor I: a biologic maturation indicator. *American Journal of Orthodontics and Dentofacial Orthopedics*.

[18] Sinha P., Trehan M., Sharma S. (2014). Assessment of skeletal maturity by correlating insulin like growth factor-1 with hand-wrist radiographs: an in vivo study. *The Journal of Indian Orthodontic Society*.

[19] Perinetti G., Franchi L., Castaldo A., Contardo L. (2012). Gingival crevicular fluid protein content and alkaline phosphatase activity in relation to pubertal growth phase. *Angle Orthodontist*.

[20] Tarvade S. M., Ramkrishna S., Sarode S. (2015). Salivary alkaline phosphatase-a biochemical marker for growth prediction. *Indian Journal Of Basic And Applied Medical Research*.

[21] Hegde S., Revankar A., Patil A. (2018). Identification of bone-specific alkaline phosphatase in saliva and its correlation with skeletal age. *Indian Journal of Dental Research*.

[22] Irham F., Bahirrah S. (2018). The level of alkaline phosphatase in saliva as biomarker for pubertal growth phase. *Advances in Health Science Research*.

[23] Navazesh M. (1993). Methods for collecting saliva. *Annals of the New York Academy of Sciences*.

[24] Perinetti G., Baccetti T., Contardo L., Di Lenarda R. (2011). Gingival crevicular fluid alkaline phosphatase activity as a non-invasive biomarker of skeletal maturation. *Orthodontics and Craniofacial Research*.

[25] Smith T. J., McKenna C. M. (2013). A comparison of logistic regression pseudo R2 indices. *Multiple Linear Regression Viewpoints*.

[26] Mokhtarian P. L. (2016). Discrete choice models’ *ρ*2: a reintroduction to an old friend. *Journal of choice modelling*.

[27] Agresti A. (2002). *Categorical Data Analysis*.

[28] Wijaya H., Kusdhany L. S., Redjeki S., Soegiharto B. M. (2019). Salivary bone-specific alkaline phosphatase as predictor of puberty phase. *Journal of International Dental and Medical Research*.

[29] Tang F. H., Chan J. L. C., Chan B. K. L. (2019). Accurate age determination for adolescents using magnetic resonance imaging of the hand and wrist with an artificial neural network-based approach. *Journal of Digital Imaging*.

[30] World Health Organization (2001). *Biomarkers in risk assessment: validity and validation-environmental health criteria 222*.

[31] Sinha M., Tripathi T., Rai P., Gupta S. K. (2016). Serum and urine insulin-like growth factor-1 as biochemical growth maturity indicators. *American Journal of Orthodontics and Dentofacial Orthopedics*.

[32] Shetty S. R., Al-Bayati S., Hamed M. S., Abdemagyd H. (2017). Salivary alkaline phosphatase and oral health: a review. *Italian Journal of Dental Medicine*.

[33] Pellegrini G. G., Gonzales Chaves M. M. S., Fajardo M. A., Ponce G. M., Toyos G. I., Lifshitz F. (2012). Salivary bone turnover markers in healthy pre- and postmenopausal women: daily and seasonal rhythm. *Clinical Oral Investigations*.

[34] Soegiharto B. M., Cunningham S. J., Moles D. R. (2008). Skeletal maturation in Indonesian and white children assessed with hand-wrist and cervical vertebrae methods. *American Journal of Orthodontics and Dentofacial Orthopedics*.

[35] Arciniega Ramos N. A., Ballesteros Lozano M., Meléndez Ocampo A. (2013). Comparative analysis between dental, skeletal and chronological age. *Revista Mexicana de Ortodoncia*.

[36] Baidas L. (2012). Correlation between cervical vertebrae morphology and chronological age in Saudi adolescents. *King Saud University Journal of Dental Sciences*.

[37] Safavi S. M., Beikaii H., Hassanizadeh R., Younessian F., Baghban A. A. (2015). Correlation between cervical vertebral maturation and chronological age in a group of Iranian females. *Dental research journal*.

[38] Macha M., Lamba B., Avula J. S. S., Muthineni S., Margana P. G. J. S., Chitoori P. (2017). Estimation of correlation between chronological age, skeletal age and dental age in children: a cross-sectional study. *Journal of Clinical and Diagnostic Research: JCDR*.

[39] Alhazmi N., Trotman C. A., Finkelman M., Hawley D., Zoukhri D., Papathanasiou E. (2019). Salivary alkaline phosphatase activity and chronological age as indicators for skeletal maturity. *Angle Orthodontist*.

